# Alcohol consumption and risk of incident heart failure in older men: a prospective cohort study

**DOI:** 10.1136/openhrt-2015-000266

**Published:** 2015-08-11

**Authors:** S Goya Wannamethee, Peter H Whincup, Lucy Lennon, Olia Papacosta, A Gerald Shaper

**Affiliations:** 1 Department of Primary Care and Population Health, University College London, London, UK; 2 Department of Population Health Sciences and Education, St George's, University of London, London, UK

**Keywords:** HEART FAILURE

## Abstract

**Aims:**

Light-to-moderate drinking has been associated with reduced risk of heart failure (HF). We have examined the association between alcohol consumption and incident HF in older British men.

**Methods and results:**

Prospective study of 3530 men aged 60–79 years with no diagnosed HF or myocardial infarction (MI) at baseline and followed up for a mean period of 11 years, in whom there were 198 incident HF cases. Men were divided into 6 categories of alcohol consumption: none, <1, 1–6, 7–13, 14–34 and ≥35 drinks/week. There was no evidence that light-to-moderate drinking is beneficial for risk of HF. Heavy drinking (≥35 drinks/week) was associated with significantly increased risk of HF. Using the large group of men drinking 1–6 drinks/week as the reference group, the relative HRs (95% confidence interval) for HF adjusted for age, lifestyle characteristics, blood pressure, atrial fibrillation and renal dysfunction were 0.97 (0.59 to 1.63), 1.39 (0.86 to 2.25), 1.00, 0.94 (0.64 to 1.43), 1.16 (0.78 to 1.71) and 1.91 (1.02 to 3.56) for the 6 alcohol groups, respectively. The increased risk associated with heavy drinking was attenuated after adjustment for N-terminal pro-brain natriuretic peptide (NT-proBNP) (HR=1.43 (0.76 to 1.69)). Stratified analysis showed heavy drinking was associated with increased HF risk only in those with ECG evidence of myocardial ischaemia.

**Conclusions:**

There was no evidence that light-to-moderate drinking is beneficial for the prevention of HF in older men without a history of an MI. Heavier drinking (≥5 drinks/day), however, was associated with increased risk of HF in vulnerable men with underlying myocardial ischaemia.

**Key messages**

**What is already known about this subject?**

- Light-to-moderate drinking is associated with reduced risk of heart failure (HF) in middle-aged populations. High levels of alcohol, usually more than 90 g/day (>9 standard UK drinks), and alcohol abuse can result in alcoholic cardiomyopathy leading to HF. Less is known about the influence of alcohol on HF in older adults in whom incident HF is high, and the level of alcohol intake which increases HF risk is uncertain.

**What does this study add?**

- We have examined the influence of light-to-moderate drinking and heavy drinking on risk of incident HF in an older population in whom HF is often not preceded by a myocardial infarction(MI), and assessed the effects of alcohol on N-terminal pro-brain natriuretic peptide (NT-proBNP) (marker of cardiac dysfunction). The study does not provide evidence that light-to-moderate drinking is protective in older adults, and that drinking 5 or more drinks is associated with significantly increased risk of HF compared with lighter drinkers in those with underlying ischaemia.

**How might this impact on clinical practice?**

- Heavier drinking (5 or more drinks/day), particularly in those with underlying ischaemia, should be avoided. There is no evidence for a beneficial effect of light-to-moderate drinking to prevent HF in men aged over 60 years who have no history of an MI, although there was also no evidence of harm from light-to-moderate drinking in those without MI.

## Introduction

Heart failure (HF) is a major and increasingly important public health problem in older people and is associated with considerable hospitalisation and mortality.^1^ Although it is well documented that high levels of alcohol intake (generally >90 g/day alcohol or more than 9 UK standard drinks) and alcohol abuse can result in alcoholic cardiomyopathy leading to HF,^2–4^ a meta-analysis and several prospective studies, predominantly from the USA and mostly conducted in middle-aged populations, have suggested that light-to-moderate drinking may be beneficial for risk of HF.^5–12^ However, this pattern was not observed in a large Finnish study^13^ and in the SAVE trial,^14^ and some studies suggest that no beneficial effect on incident HF is seen with light or moderate drinking (1–2 drinks/day) when HF is not preceded by a myocardial infarction (MI).^11^ This suggests that the lower risk of HF associated with light-to-moderate drinking may be mediated through beneficial effects of alcohol on coronary heart disease (CHD).^11^ However, few studies have examined the association between alcohol drinking and risk of HF in older adults, in whom the occurrence of HF with preserved ejection fraction is more common than HF with reduced ejection fraction^15^ and in whom HF is less likely to be associated with CHD.^16^ Moreover, in most population-based cohort studies which have examined the association between alcohol consumption and HF risk, heavier drinking (>3 drinks/day) is under-represented and the level of alcohol intake which is associated with increased HF risk is less certain although prospective population-based studies have suggested that the risk of developing atrial fibrillation, a major risk factor for HF, increases significantly with 5 or more drinks/day^17^ and at an even lower threshold (>3 drinks/day) in those with cardiovascular (CV) disease (CVD).^18^ We have, therefore, examined the association between alcohol consumption and incident HF in a prospective study of older British men aged 60–79 years with particular focus on whether heavy drinking is associated with increased HF risk compared with lighter drinking.

## Participants and methods

The British Regional Heart Study is a prospective study involving 7735 men aged 40–59 years drawn from one general practice in each of 24 British towns, who were screened between 1978 and 1980.^19^ The population studied was socioeconomically representative of British men and comprises predominantly white Europeans (>99%). In 1998–2000, all surviving men, now aged 60–79 years, were invited for a 20th year follow-up examination. Ethical approval was obtained from all relevant local research ethics committees. All men completed a mailed questionnaire providing information on their lifestyle and medical history, had a physical examination and provided a fasting blood sample. The samples were frozen and stored at −20°C on the day of collection and transferred in batches for storage at −70°C until analysis, which was carried out after no more than one freeze–thaw cycle. The 12-lead ECGs were recorded using a Siemens Sicard 460 instrument and were analysed using Minnesota Coding definitions at the University of Glasgow ECG core laboratory.^20^ The men were asked whether a doctor had ever told them that they had angina, MI (heart attack, coronary thrombosis), HF or stroke; details of their medications were recorded at the examination. In total, 4252 men (77% of survivors) presented for examination. Blood measurements were available for 4045 men. We excluded 117 men with prevalent HF and a further 395 men with a history of doctor-diagnosed MI, and 3 men with no information on alcohol intake. We excluded men with prevalent MI because these men are at particularly high risk of HF and are particularly likely to receive advice to change their lifestyle, including alcohol intake. The analyses are thus based on 3530 men.

### CV risk factors at 1998–2000

Anthropometric measurements, including body weight and height, were carried out with participants standing in light clothing without shoes. Details of measurement and classification methods for smoking status, physical activity, social class, blood pressure, blood lipids and forced expiratory volume in 1 s (FEV_1_) in this cohort have been described.^21–23^ Prevalent diabetes included men with a diagnosis of diabetes and men with fasting blood glucose ≥7 mmol/L. Predicted glomerular filtration rate (eGFR) was estimated from serum creatinine;^24^ GFR=186*((creatinine)**−1.154)*((age)**−0.203). N-terminal pro-brain natriuretic peptide (NT-proBNP) was determined using the Elecsys 2010 electrochemiluminescence method (Roche Diagnostics, Burgess Hill, UK).^23^ NT-proBNP measurements were performed at baseline, and not in close relation to incident events. Electrocardiographic left ventricular hypertrophy (LVH) was defined according to relevant Minnesota Codes (codes 3.1 or 3.3). Atrial fibrillation was defined according to Minnesota Codes 8.3.1 and 8.3.3. Evidence of myocardial ischaemia on ECG was based on Minnesota Codings 1.1–1.3 (definite, probable or possible MI) or 4.1–4.4 and 5.1–5.3 (definite, probable or possible myocardial ischaemia). In total, 102 men had silent MI on ECG with no history of a doctor diagnosis of MI; these men were included in the study.

### Alcohol intake

The men were asked to describe their current frequency of drinking (daily, most days, weekend only, occasional, special occasions only or none) and were asked to estimate the number of alcoholic drinks during an average week. The men were classified into six groups according to their reported weekly intake: none (n=344), <1 (n=338), 1–6 (n=1259), 7–13 (n=712), 14–34 (n=740) and ≥35 drinks/week (n=137). One UK drink=10 g alcohol. Light-to-moderate drinking refers to those drinking up to 34 drinks/week. Heavy drinking is defined as drinking ≥35 drinks/week (5 or more drinks daily). To achieve sufficient numbers and because previous report suggest that risk of atrial fibrillation, a major risk factor for HF, is only elevated at levels of 5 drinks or more a day, we have used ≥35 drinks as the threshold for heavy drinking. We have used the large group of 1–6 drinks/week as the reference group for comparison purposes. The alcohol questionnaire used in this study has been validated using 25 biochemical and haematological measurements on a single blood sample which indicated that the reported levels of alcohol consumption were valid on a group basis.^25^

### Follow-up

All men have been followed up from initial examination (1978–1980) for CV morbidity and development of diabetes and follow-up has been achieved for 99% of the cohort.^26^ In the present analyses, all-cause mortality and morbidity events are based on follow-up from the rescreening examination in 1998–2000 to July 2010, a mean follow-up period of 11 years (range 10–12 years). Information on death was collected through the established ‘tagging’ procedures provided by the National Health Service registers. Fatal CHD events were defined as death with CHD (International Classification of Diseases (ICD), Ninth Revision, codes 410–414) as the underlying code. A non-fatal MI was diagnosed according to WHO criteria.^27^ Evidence of non-fatal MI and HF was obtained by ad hoc reports from general practitioners supplemented by biennial reviews of the patients’ practice records (including hospital and clinic correspondence) through to the end of the study period. Possible or probable cases were not included in incident CHD cases. Incident non-fatal HF was based on a confirmed doctor diagnosis of HF from primary care records and where possible, verified using details of available clinical information from primary and secondary care records (including symptoms, signs, investigations, treatment response) to ensure that the diagnosis was consistent with current recommendations on HF diagnosis.^28^ These data were available for 160 of the 194 non-fatal cases (82%). The incidence and determinants of HF cases identified using this process has already been reported and are consistent with results from other studies.^22^ ^23^ Incident HF included incident non-fatal HF (194 cases) as well as death from HF (4 cases) as the underlying cause (ICD, Ninth revision, code 428).

### Statistical methods

The χ^2^ tests and the analysis of variance were used to assess the difference in baseline characteristics between the six alcohol groups. Cox’s proportional hazards model was used to assess the multivariate-adjusted HR (relative risk) by alcohol categories. The large group of men drinking 1–6 drinks/week was used as the reference group. The proportional hazards assumption was examined using time-varying covariates, calculating interactions of predictor variables and a function of survival time and including these in the models. Examination of time-varying covariates indicated no violation of the proportionality assumption in the sample. In multivariate analyses, smoking (never-smokers, long-term ex-smokers (>15 years), recent ex-smokers (<15 years) and current smokers), social class (manual vs non-manual), physical activity (4 groups), diabetes (yes/no), use of antihypertensive treatment (yes/no), prior stroke (yes/no), diagnosed angina (yes/no), LVH (yes/no), renal dysfunction (yes/no), and atrial fibrillation (yes/no) were fitted as categorical variables. Systolic blood pressure, body mass index, FEV_1_ and NT-proBNP were fitted continuously. To evaluate whether alcohol predicted HF independent of incident MI (ie, those who developed MI during follow-up), we adjusted for incident non-fatal MI, fitting this as a time-dependent covariate.

## Results

During the mean follow-up period of 11 years, there were 198 incident HF cases (rate 5.9/1000 person-years) and 336 major CHD events in the 3530 men with no doctor diagnosis of HF or MI at baseline (ie, prevalent MI).

Table 1 shows the baseline characteristics by levels of alcohol consumption. Overall non-drinkers and heavy drinkers (≥35 drinks/week) tended to have the most adverse CV risk factors. In particular the non-drinkers were the oldest group and had the highest prevalence rates of physical inactivity, manual workers, stroke, atrial fibrillation and renal dysfunction. They also had the highest mean levels of NT-proBNP. Since NT-proBNP is strongly associated with age (r=0.40; p<0.0001), adjustment for age attenuated the increased NT-proBNP levels in non-drinkers and increased the mean levels in heavy drinkers, who then showed the highest NT-proBNP levels. Men drinking <1 drink/week in general had characteristics close to those of non-drinkers. There was an inverse association between alcohol intake and the percentage of men who developed CHD during follow-up.

**Table 1:** Baseline characteristics by alcohol consumption in men without prevalent diagnosed MI or HF

			Current alcohol intake (drinks/week)				
	Non-drinker (N=344)	<1 (N=338)	1–6 (N=1259)	7–14 (N=712)	15–34 (N=740)	≥35 (N=137)	p difference
Age (years)	69.7 (5.5)	68.8 (5.3)	68.6 (5.5)	68.5 (5.5)	67.8 (5.2)	67.4 (5.2)	<0.0001
BMI (kg/m^2^)	26.8 (4.3)	26.7 (3.7)	26.6 (3.4)	26.8 (3.4)	27.1 (3.6)	27.0 (3.8)	0.20
Obese (%)	15.4	15.0	14.5	16.7	17.0	20.4	0.41
Current smokers (%)	18.7	16.3	10.0	11.4	12.9	22.2	<0.0001
Inactive (%)	45.8	39.4	32.1	27.0	31.0	34.1	<0.0001
Manual workers (%)	68.0	64.6	52.1	50.8	43.2	56.2	<0.0001
Past >6 drinks/day drinkers (%)	3.2	2.0	3.7	11.6	23.9	66.4	<0.0001
Stroke (%)	8.1	2.7	4.2	4.4	5.6	2.9	0.008
Atrial fibrillation (%)	3.8	3.8	2.8	3.3	3.6	1.5	0.64
Angina (%)	11.6	10.0	8.3	7.3	8.7	3.7	0.05
Diabetes (%)	14.5	14.8	10.7	12.2	12.7	15.3	0.24
On BP- lowering treatment (%)	29.9	31.6	28.8	26.9	27.8	26.3	0.70
LVH (%)	9.0	6.2	8.0	7.4	6.8	9.5	0.60
ECG evidence of ischaemia (%)	27.1	20.8	21.9	22.0	21.9	25.3	0.08
FEV_1_ (L)	2.47 (0.6)	2.58 (0.7)	2.67 (0.7)	2.61 (0.6)	2.64 (0.6)	2.53 (0.6)	<0.0001
Heart rate (bpm)	67.1 (12.5)	67.0 (13.4)	64.6 (12.2)	65.4 (12.4)	66.6 (13.2)	69.1 (13.6)	<0.0001
SBP (mm Hg)	148.4 (22.7)	147.3 (23.2)	149.4 (24.7)	150.0 (24.0)	152.5 (22.9)	157.4 (22.9)	<0.0001
HDL-C (mmol/L)	1.25 (0.3)	1.25 (0.3)	1.28 (0.3)	1.36 (0.3)	1.42 (0.4)	1.61 (0.4)	<0.0001
GGT (IU/L)*	25.8 (18–33)	25.2 (17–34)	26.3 (18–35)	29.1 (19–37)	33.8 (25–46)	45.6 (27–31)	<0.0001
eGFR (mL/min/1.73 m^2^)	71.1 (13.9)	71.9 (14.5)	71.7 (12.3)	72.7 (12.6)	73.9 (11.4)	75.0 (12.2)	<0.0001
Renal dysfunction (%)	20.4	15.5	15.7	12.7	10.8	13.1	0.0004
NT-proBNP (pg/mL)*	105.6 (45–207)	88.2 (41–183)	85.6 (41–165)	87.4 (45–157)	86.5 (42–159)	101.5 (51–198)	0.04
(Age-adjusted)	(95.6)	(87.4)	(85.6)	(85.6)	(89.1)	(111.0)	0.07
Developed CHD (%)	13.1	11.5	9.6	8.0	8.5	8.0	0.08

Mean (SD) unless specified.

Data on alcohol not available in three men.

*Geometric mean and IQR.

BMI, body mass index; BP, blood pressure; CHD, coronary heart disease; eGFR, estimated glomerular filtration rate; FEV_1_, forced expiratory volume in 1 s; GGT, γ-glutamyl-transpeptidase; HDL-C, high-density lipoprotein cholesterol; HF, heart failure; LVH, left ventricular hypertrophy; MI, myocardial infarction; NT-proBNP, N-terminal pro-brain natriuretic peptide; SBP, systolic blood pressure.

**Figure 1:**
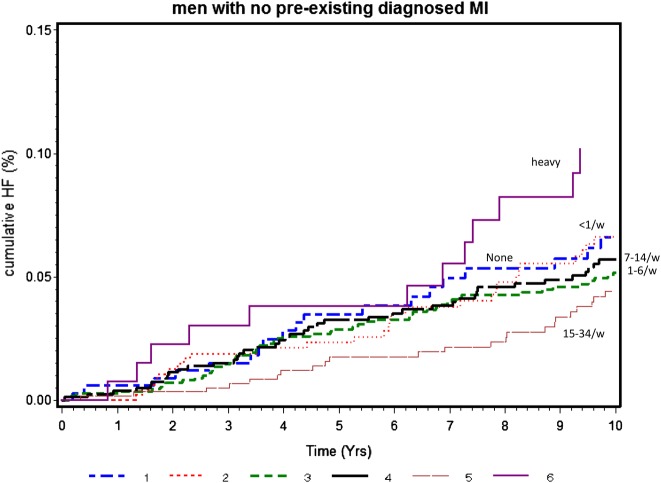
Kaplan-Meier curve of cumulative heart failure (HF) incidence by alcohol intake in men with no diagnosed myocardial infarction (MI). Log rank test p=0.14. Drinks/week 1=none; 2=<1; 3=1–6; 4=7–14; 5=15–34; 6=≥35 (heavy).

Figure 1 shows the Kaplan-Meier curve of the cumulative incidence of HF by levels of alcohol consumption in men without a doctor diagnosis of MI. Heavy drinkers showed the highest cumulative incidence of HF followed by those drinking none or <1 drink/week. Incidence rates and relative HR for HF at different levels of alcohol consumption, using the large group of men consuming (1–6 drinks/week) as the reference group, are shown in table 2. In age-adjusted analyses, non-drinkers and infrequent drinkers showed higher risk, albeit non-significantly, compared with those drinking 1–6 drinks/week. Adjustment for CV risk factors (model 3) attenuated the increased HF risk in non-drinkers and these showed similar risk to both groups of light drinkers (1–6 and 7–13 drinks/week groups). Heavy drinkers showed significantly increased HF risk, but this persisted even after adjustment for these CV risk factors. Further adjustments for FEV_1_ (model 4) and in particular NT-proBNP (model 5) attenuated the increased relative risk of HF associated with heavy drinking. Further adjustment for incident MI strengthened the association in heavy drinkers, but it remained non-significant. Exclusion of all men who had ever reported heavy drinking (>6 drinks/day since entry to the study at 40–59 years) in model 2 made little difference to the findings. We carried out further analysis separating the 21–34 drinks/week from the 15–34 drinks/week category. The adjusted relative risk (95% confidence interval) were 1.00 (0.60 to 1.67), 1.43 (0.88 to 2.31), 1.00, 1.02 (0.67 to 1.57), 1.48 (0.94 to 2.34), 0.57 (0.25 to 1.31) and 1.80 (0.96 to 3.36) for the seven groups: none, <1, 1–6, 7–14, 15–20, 21–34 and ≥35 drinks/week, respectively.

**Table 2:** Heart failure rates/1000 person-years and adjusted HR for heart failure according to alcohol consumption in men with no pre-existing diagnosed myocardial infarction (MI) or heart failure

		Alcohol intake (drinks/week)				
	Non-drinker (N=344)	<1 (N=338)	1–6 (N=1259)	7–14 (N=712)	15–34 (N=740)	≥35 (N=137)
Rates/1000 person-years (n)	6.9 (21)	7.9 (25)	5.2 (64)	5.0 (34)	5.9 (42)	9.4 (12)
Age-adjusted	1.19 (0.71 to 1.92)	1.54 (0.96 to 2.48)	1.00	0.98 (0.65 to 1.49)	1.17 (0.80 to 1.73)	2.04 (1.10 to 3.77)
Model 1	0.98 (0.59 to 1.63)	1.43 (0.89 to 2.32)	1.00	0.94 (0.62 to 1.43)	1.15 (0.78 to 1.70)	1.90 (1.02 to 3.54)
Model 2	1.01 (0.61 to 1.61)	1.49 (0.92 to 2.32)	1.00	0.95 (0.63 to 1.45)	1.19 (0.81 to 1.76)	1.89 (1.01 to 3.52)
Model 3	0.97 (0.59 to 1.63)	1.39 (0.86 to 2.25)	1.00	0.94 (0.64 to 1.43)	1.16 (0.78 to 1.71)	1.91 (1.02 to 3.56)
Model 4	0.94 (0.57 to 1.58)	1.44 (0.86 to 2.29)	1.00	0.93 (0.58 to 1.36)	1.14 (0.76 to 1.67)	1.80 (0.96 to 3.36)
Model 5	0.92 (0.55 to 1.56)	1.28 (0.75 to 2.10)	1.00	0.93 (0.61 to 1.43)	1.14 (0.76 to 1.69)	1.43 (0.76 to 1.69)
Model 6	0.93 (0.55 to 1.57)	1.38 (0.84 to 2.29)	1.00	1.02 (0.66 to 1.56)	1.22 (0.82 to 2.82)	1.56 (0.81 to 3.00)
Model 2 and exclusion of past heavy drinkers (>6 drinks/day)	0.95 (0.57 to 1.58)	1.38 (0.85 to 2.24)	1.00	0.99 (0.65 to 1.51)	1.15 (0.76 to 1.74)	1.83 (0.98 to 3.40)

Model 1: adjusted for age, smoking, body mass index, social class, prevalent stroke, diabetes and angina.

Model 2: Model 1+left ventricular hypertrophy, antihypertensive drugs and systolic blood pressure.

Model 3: Model 2+atrial fibrillation and renal dysfunction.

Model 4: Model 3+forced expiratory volume in 1 s.

Model 5: Model 4+N-terminal pro-brain natriuretic peptide (NT-proBNP).

Model 6: Model 5+incident MI.

We also examined the association between alcohol and HF stratified by age (<70 vs ≥70 years) using non-drinkers and men drinking <1 drink/week combined as the reference group. The number of heavy drinkers (≥35 drinks/week) in the oldest age group (n=39) was too few to examine separately. In men <70 years (N=2183;86 cases), the relative risk (95% CI) adjusted for factors in model 2 (table 2) using non-drinkers and <1 drink/week combined as the reference group were 1.00, 0.89 (0.46 to 1.75), 0.67 (0.30 to 1.53), 2.37 (1.13 to 4.98) and 2.67 (1.15 to 6.19) for none or <1, 1–6, 7–14, 15–34 and ≥35 drinks/week, respectively. No association was seen in the older age group (N=1347; 112 cases). The relative risks in those ≥70 years were 1.00, 0.84 (0.51 to 1.37), 0.91 (0.52 to 1.61) and 0.79 (0.42 to 1.48) for none/<1, 1–6, 7–14 and 15–34 drinks/week groups, respectively.

Since NT-proBNP is a marker of cardiac damage, we further examined the association between alcohol intake and HF risk separately in men with and without ECG evidence of myocardial ischaemia (table 3). To achieve sufficient numbers, the non-drinkers and those reporting <1 drink/week were combined, and the 7–14 and 14–34 drinks/week groups were combined, creating four alcohol groups overall. Evidence of myocardial ischaemia was present in about 22% of the men with no diagnosed MI. Heavy drinking was associated with increased risk of HF only in those with ECG evidence of myocardial ischaemia and this remained even after adjustment for NT-proBNP and exclusion of men with silent MI (n=102). No association was seen at all between alcohol intake and HF risk in those with no evidence of myocardial ischaemia. Exclusion of heavy drinkers made little difference to the findings in men with no myocardial ischaemia. In those with ischaemia, those drinking <1 drink/week showed higher risk compared with those drinking 1–6 drinks/week, but the difference was not significant. We carried out a sensitivity analysis restricting the analyses to the 160 validated cases. This did not materially change the results. The adjusted relative risks (model 1) for the four alcohol groups in those with no ischaemia were 1.02 (0.59 to 1.78), 1.00, 1.14 (0.72 to 1.78) and 1.22 (0.43 to 3.48) respectively, and 1.61 (0.80 to 3.23), 1.00, 1.19 (0.63 to 2.24) and 3.54 (1.36 to 9.23) in those with ischaemia, respectively.

**Table 3:** Age-adjusted mean NT-proBNP and adjusted HR for heart failure according to alcohol consumption in men with and without ECG evidence of ischaemia

	Alcohol intake			
	<1-week	1–6/week	7–34/week	≥35/week
	Men without ECG evidence of myocardial ischaemia			
N (cases)	519 (25)	983 (43)	1142 (48)	102 (4)
Age-adjusted mean NT-proBNP	78.3	73.7	75.2	88.2
Model 1	0.94 (0.56 to 1.57)	1.00	1.01 (0.67 to 1.53)	0.99 (0.55 to 2.79)
Model 2	0.84 (0.49 to 1.43)	1.00	1.02 (0.67 to 1.54)	0.83 (0.29 to 2.36)
Model 2 (exclude past heavy drinkers)	0.92 (0.52 to 1.64)	1.00	0.97 (0.63 to 1.51)	0.78 (0.27 to 2.21)
	Men with ECG evidence of myocardial ischaemia			
N (cases)	163 (21)	276 (21)	310 (28)	35 (8)
Age-adjusted mean NT-proBNP	148.4	145.4	148.0	219.2
Model 1	1.60 (0.85 to 3.02)	1.00	1.11 (0.62 to 2.00)	3.53 (1.52 to 8.24)
Model 2	1.62 (0.84 to 3.11)	1.00	1.13 (0.62 to 2.03)	3.11 (1.27 to 7.57)
Model 2 (excluding silent MI)	1.43 (0.69 to 2.95)	1.00	1.02 (0.54 to 1.90)	3.01 (1.15 to 7.87)
Model 2 (exclude past heavy drinkers)	1.85 (0.88 to 3.88)	1.00	1.32 (0.68 to 2.54)	3.40 (1.36 to 8.50)

Model 1: adjusted for age, smoking, BMI, social class, prevalent stroke, diabetes, angina, LVH, antihypertensive drugs, systolic blood pressure, atrial fibrillation, renal dysfunction and FEV_1_.

Model 2: Model 1+NT-proBNP.

Past heavy drinkers=those ever reporting drinking ≥6 drinks/day.

BMI, body mass index; FEV_1_, forced expiratory volume in 1 s; LVH, left ventricular hypertrophy; MI, myocardial infarction; NT-proBNP, N-terminal pro-brain natriuretic peptide.

## Discussion

In this study of older British men, there was no evidence that light-to-moderate drinking (up to 4 drinks/day) was associated with a beneficial effect on HF risk overall; non-drinkers showed similar risk to light/moderate drinkers (up to 34 drinks/week). Heavy drinking (≥35 drinks/week or ≥5 drinks/day), however, was associated with a significantly increased risk of HF compared with those drinking 1–6 drinks/week, which was largely seen in those with ECG evidence of myocardial ischaemia. Our findings extends those of previous studies by examining the effects of both light-to-moderate drinking as well as heavy drinking on risk of HF and the role of NT-proBNP, a marker of cardiac damage, and underlying myocardial ischaemia.

### Light-to-moderate drinking and HF

Previous studies mainly from the USA have suggested that light-to-moderate drinking is associated with a beneficial effect on HF.^5–12^ However, recent findings from a large Finnish cohort showed no beneficial effect of light drinking on HF similar to the current findings.^13^ Most of these previous studies were conducted in middle-aged populations. In one study it was suggested that the benefit of light-to-moderate drinking is due to its effect on reducing CHD, which in turn is associated with reduced rates of HF rather than a direct beneficial effect of alcohol on HF risk.^11^ In the US Kaiser Permanente study, light-to-moderate drinking was associated with reduced risk of HF associated with coronary artery disease (CAD), but no benefit was seen for light-to-moderate drinking and the risk of non-CAD-related HF, and heavy drinking >3 drinks/day increased risk of non-CAD-related HF.^7^ Older patients with HF differ from younger patients in that a higher proportion of older patients with HF have HF with preserved ejection fraction.^15^ These patients are less likely to have CHD and more likely to have hypertension and atrial fibrillation.^16^ A high proportion of men without a history of a doctor diagnosis of an MI in this study who developed HF did not develop an MI before developing HF (85%), which would explain the difference in findings between this and the younger US cohorts. In contrast, CHD is the predominant risk factor for HF with reduced ejection fraction, which is more common in younger adults.^16^ This may explain the overall lower risk of HF associated with light-to-moderate drinking seen in many of the previous studies which have been conducted in younger populations. The association between alcohol and HF in older adults has been less well studied. In the Cardiovascular Health Study of men aged >65 years, a weak but non-significant inverse association was seen between light-to-moderate alcohol intake and HF in those without CVD.^8^

The magnitude of the health effects of alcohol is dependent on the base group used; non-drinkers and infrequent drinkers are usually not an appropriate group to estimate the effects of alcohol.^29^ For comparison purposes with other studies, when non-drinkers and infrequent drinkers were used as the comparison group, we showed a weak and non-significant protective effect of light-to-moderate drinking on HF risk in this older population. Our findings are not dissimilar to that seen in the Cardiovascular Health Study. However, the inconsistent findings that non-drinkers and the infrequent drinkers had different risk patterns for HF does not provide convincing evidence for a protective effect of alcohol on HF in older men. This is in contrast to CHD events where there was a clear inverse relation between alcohol drinking and the percentage of men who developed an MI in this study, as expected. Moreover, there was no association at all between alcohol intake and HF in the large group of men with no evidence of myocardial ischaemia. While we cannot exclude the possibility that light-to-moderate drinking may have some protective effect in men with ischaemia, we cannot confirm this in our study.

### Heavy drinking, NT-proBNP and HF risk

It is well established that excessive alcohol intake, usually defined as >90 g/day (more than 9 standard UK drinks), can lead to cardiomyopathy,^3^ which in turn leads to left ventricular dysfunction and HF.^2^ Most studies on heavy drinking and cardiomyopathy have been conducted in alcoholic patients, in whom detectable changes in cardiac structure are seen in those who report drinking >90 g/day.^3^ However, few studies have been able to examine the impact of heavier drinking below this threshold on HF risk in the general population because of the low prevalence of heavy drinkers in these study populations. Most population studies in the US which have investigated the association between alcohol and incident HF have focused on the benefits of light drinking and no increased risk is seen with heavier drinking, usually defined as just >2 drinks/day. However, in the US Kaiser Permanente study, heavy drinking defined as >3 drinks/day was shown to be associated with non-CAD-related HF.^7^ In a Swedish population study, men who reported history of alcohol abuse showed increased risk of HF.^30^ We have shown that risk of HF is increased in men consuming 5 or more drinks/day, the majority of whom reported drinking <8 drinks/day. These findings are in keeping with those from the Copenhagen Study which showed that risk of *incident* atrial fibrillation, a major risk factor for HF, is only increased at levels of 5 drinks or more.^17^ We observed no association between alcohol intake and *prevalent* atrial fibrillation. However, this may be due to the fact that many men with atrial fibrillation are likely to develop stroke or CVD, and have reduced their intake as a result of developing CVD.

NT-proBNP, a peptide released from myocardium in response to ventricular wall stress and dysfunction, is a marker of myocardial damage and subclinical cardiac function, and is strongly related to incident atrial fibrillation and HF.^23^ ^31^ A recent report has shown NT-proBNP to be increased in heavy drinkers in a population-based study^32^ which is consistent with our findings. We have shown that the effect of heavy drinking on incident HF risk was to a large extent associated with NT-proBNP. This may reflect a direct toxic effect of alcohol on the myocardium with consequent effects on cardiac function and ventricular wall stress, as has been suggested.^32^ Further subsidiary analysis showed that heavy drinking (≥5 drinks/day) increased risk only in those with ECG evidence of myocardial ischaemia and this was only partly due to raised NT-proBNP. Thus, regular heavy drinking appears to have adverse effects on HF risk, particularly in the presence of myocardial ischaemia where it may aggravate the myocardial damage or dysfunction leading to HF.

### Limitations

There are several limitations in this study. The current findings are based on doctor-diagnosed HF, which is likely to underestimate the true incidence of HF in this study population; however, these diagnoses are usually supported by evidence from hospital admissions and hospital attendances investigation. These men who were survivors of an ongoing cohort study and attended the re-examination may have been healthier than the general older population which might have also affected the absolute incident rates of HF. However, this should not have affected the associations between alcohol and HF risk; our previous reports on HF predictors, such as obesity and NT-proBNP, in this cohort have generally accorded with prior data and therefore, suggest external validity for our findings.^22^ ^23^ Echocardiographic measurements were not carried out in the present study and we were not able to differentiate HF with reduced ejection fraction and HF with preserved ejection fraction. We did not have information on incident atrial fibrillation to assess the possible mediating role of atrial fibrillation. Although we did not see a significant effect for light-to-moderate drinkers, the number of men drinking <1 week in this study was small and the CIs do not exclude such an effect completely because of the size and power of the study. Although we excluded past heavy drinkers, higher levels of alcohol consumption may have been under-reported, leading to a misclassification of heavier drinkers into lower consumption categories. Such a misclassification may have attenuated any protective effects present among true light-to-moderate drinkers. Finally, it was based on an older, predominantly white, male population of European origin, so that the results cannot be generalised directly to women, or to younger populations or other ethnic groups.

## Conclusion

In this study of older British men, there is no evidence that light-to-moderate drinking is beneficial for the prevention of HF in men without MI; heavier drinking ≥5 drinks/day is associated with increased risk of HF, particularly in men with underlying myocardial ischaemia.
